# A Case of Giant Cell Arteritis With a Normal Erythrocyte Sedimentation Rate (ESR) Post ChAdOx1 nCoV-19 Vaccination

**DOI:** 10.7759/cureus.25388

**Published:** 2022-05-27

**Authors:** Chenfan Xia, Rachel Edwards, Bita Omidvar

**Affiliations:** 1 Department of Medicine, Frankston Hospital, Melbourne, AUS; 2 Department of Medicine, Casey Hospital, Melbourne, AUS; 3 Department of Rheumatology, Frankston Hospital, Melbourne, AUS

**Keywords:** normal esr, esr, temporal artertitis, covid-19 vaccine complication, covid-19 vaccine, covid-19, covid, inflammatory processes/inflammatory markers, gca

## Abstract

Giant cell arteritis (GCA) has been reported post the coronavirus disease 2019 (COVID-19) vaccination, especially with the mRNA vaccine. A normal erythrocyte sedimentation rate (ESR) is seen in some GCA patients.

This report describes a 68-year-old gentleman who presented with a right-sided temporal headache for three weeks, starting three to five days after his second dose of the ChAdOx1 nCoV-19 vaccine, a viral vector vaccine, which was given seven weeks post the first dose. On presentation, he developed blurred vision in the left eye, and it progressed to complete vision loss four days later. He also had episodes of blurred vision in the right eye. The blood test showed a mildly elevated C-reactive protein of 29 mg/L and a normal erythrocyte sedimentation rate (ESR) of 4 mm/hr. Optical coherence tomography showed anterior ischaemic optic neuropathy in the left eye and retinal ischemia in the right eye. Bilateral giant cell arteritis (GCA) was confirmed on temporal artery biopsy. He was treated with methylprednisolone pulse therapy followed by prednisolone. He re-presented with intermittent blurry vision in the right eye three months later. He was treated with methylprednisolone pulse therapy again, followed by prednisolone, aspirin, and tocilizumab.

This case describes a patient who developed GCA post ChAdOx1 nCoV-19 vaccination with a normal ESR. Further studies are needed to investigate this relationship as causal or incidental and the likelihood of low-level inflammatory makers in such a situation.

## Introduction

There are raising concerns that the coronavirus disease 2019 (COVID-19) vaccine may be associated with autoimmune responses, triggering autoimmune disorders such as immune-mediated thrombotic thrombocytopenia and neurological autoimmune disease [1]. Giant cell arteritis (GCA) has also been reported post the COVID-19 vaccination. Although a clear association has not been established, a recent study suggests a potential safety alert for GCA post COVID-19 vaccines, especially with the mRNA vaccine [2]. GCA is often associated with a high erythrocyte sedimentation rate (ESR). However, a normal ESR can be seen in 5-30% of GCA patients [3]. This report describes a case of biopsy-proven GCA with a normal ESR after receiving the ChAdOx1 nCoV-19 vaccine (AstraZeneca Vaxzevria), a viral vector vaccine, leading to permanent vision loss in one eye. The case was reported to the Therapeutic Goods Administration (TGA) in Australia. 

## Case presentation

A 68-year-old gentleman presented with a right-sided temporal headache for three weeks. The patient recalled the headache starting roughly three to five days after his second dose of the ChAdOx1 nCoV-19 vaccine, which was given seven weeks post the first dose. His past history included mild chronic obstructive pulmonary disease, and he was a current smoker with an 80 pack-year history.

A transient episode of blurred vision in the left eye was reported to have occurred while in the emergency department waiting room. Visual acuity was 6/6-2 in the left eye and 6/6 in the right eye. He had a normal visual field on gross examination, and there was no temporal artery tenderness. The blood test showed a normal white cell count of 8.3 × 10^9^/L (ref range: 4 to 11× 10^9^/L) and mildly elevated C-reactive protein (CRP) of 29 mg/L (ref range: 0 to 10 mg/L). Cerebral computed tomography (CT) scan and venogram were unremarkable. 

**Figure 1:**
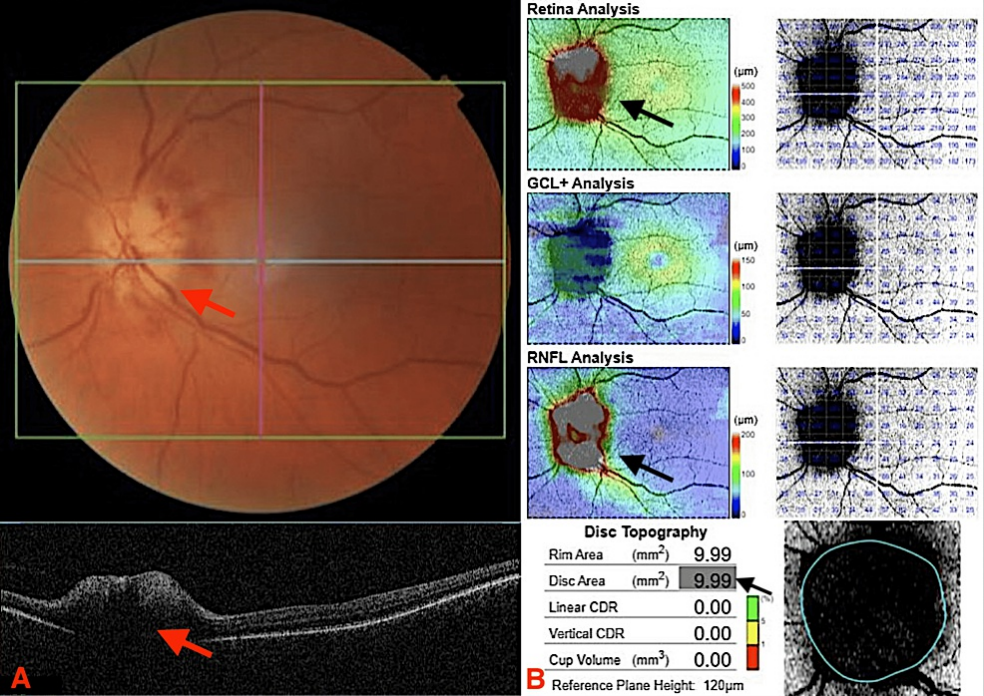
OCT showing abnormal optic disc (A) Optic disc is pale and edematous (red arrows). (B) Increased disc thickness and increased disc area (black arrows). OCT: optical coherence tomography, GCL: ganglion cell layer, RNFL: retinal nerve fibre layer, CDR: cup-to-disc ratio

Four days later, he re-presented with complete vision loss of the left eye with no light perception. Visual acuity for the right eye was 6/9-2. Optical coherence tomography (OCT) showed anterior ischaemic optic neuropathy in the left eye (Figures 1A, 1B)

He had been experiencing bilateral jaw claudication and profound lethargy, but no scalp tenderness, fever, weight loss, and no shoulder, neck or hip pain. The blood test revealed a normal ESR of 4 mm/hr (ref range: < 15 mm/hr), and CRP remained the same. Cerebral CT and angiogram from the aortic arch to vertex were normal. There was no evidence of vasculitis or acute ischemia. 

He was treated with a 60 mg dose of prednisolone, followed by four days of 1 gm intravenous methylprednisolone pulse therapy considering the significant vision loss. The headache and jaw claudication improved after the commencement of steroids. However, he reported a couple of hour-long episodes of blurred vision in the right eye on day three. 

On day four, he had 50 mg of prednisolone in the morning. OCT revealed a new finding of retinal ischaemia in the right eye, but there was no change to visual acuity. To protect the right eye vision, a fourth 1 gm dose of methylprednisolone was given in the afternoon. His prednisolone dose was increased to 65 mg daily (1 mg/kg) on day five.

Bilateral GCA was confirmed the next day on a temporal artery biopsy. He was discharged home with prednisolone 65 mg daily for one month, then 50 mg daily, trimethoprim and sulfamethoxazole three times a week for *Pneumocystis jirovecii* prophylaxis. 

He re-presented with intermittent blurry vision in the right eye three months later. He was treated with 1 gm intravenous methylprednisolone for three days, followed by prednisolone 65 mg daily, aspirin 100 mg daily, and tocilizumab 162 mg subcutaneous injection weekly. He had no new visual symptoms after being started on tocilizumab and was able to gradually reduce the prednisolone to 25 mg daily after three months. 

## Discussion

Increased incidence of GCA has been noted during the COVID-19 pandemic [4], and a case of GCA has been reported post COVID-19 infection [5], suggesting a possible connection between SARS-CoV-2 and GCA. Alterations in the immune system and infections such as the varicella-zoster virus infection are associated with increased susceptibility to GCA [6]. There are similarities between the human proteins and SARS-CoV-2 spike glycoprotein, and potential antigenic cross-reactivity between the two may increase the risk of autoimmune disease [7]. The development of autoimmune antibodies such as antinuclear antibodies (ANA) and extractable nuclear antigen antibodies (ENA) has been reported in some patients with positive SARS-CoV-2 immunoglobulin G (IgG) and immunoglobulin M (IgM) antibodies [7]. The autoimmune antibody development may apply to the COVID-19 vaccines, leading to vaccine-induced autoimmunity [7,8].

There are a few case reports of the GCA post COVID-19 vaccine, especially with mRNA vaccine. One study reported three cases [9]. Two cases were new diagnoses of polymyalgia rheumatica (PMR) and GCA after the vaccine. In the other case, a patient with pre-existing PMR had a flare post vaccines, including ChAdOx1 nCoV-19 vaccine as the first dose and mRNA vaccine as the second dose [9]. Another study described a 62-year-old woman who developed profound fatigue one or two days after the first dose of the BNT162b2 mRNA covid-19 vaccine [10]. Symptoms increased after the second dose, with weight loss, night sweat and nausea, but no headache. After seven weeks of illness, she was diagnosed with GCA on a positron emission tomography (PET) scan with a CRP of 98 mg/L [10]. Other mRNA vaccine-related case reports include a 79-year-old man who developed GCA two days after his second dose of the vaccine [11], an 82-year-old man who developed bilateral GCA with skin necrosis 10 days after his second dose of vaccine [12], a new-onset GCA in an 83-year-old woman happened 24 hours after the first dose of the vaccine [13], a 63-year-old woman who developed GCA the day after the first dose of the vaccine [14], and a 78-year-old female diagnosed with large vessel vasculitis post vaccine with symptoms that started after the first shot and worsened after the second shot [15].

There is also one case report about GCA diagnosis after the first dose of the ChAdOx1 nCoV-19 vaccine in a 70-year-old man [16]. Interestingly, his CRP was initially normal at 5.0 mg/L with an upper level of only 13.5 mg/L during his admission [16]. Considering our patient also had a normal ESR of 4 mm/hr and mildly raised CRP of 29 mg/L, this may suggest a trend towards a phenotype of GCA with low-level inflammatory markers post ChAdOx1 nCoV-19 vaccine, which may be noteworthy given that it may lead to delayed time to diagnosis and treatment with steroids [16].

## Conclusions

Increased incidence of GCA has been seen post COVID-19 vaccination, especially with the mRNA vaccine. This case described a patient who developed GCA post ChAdOx1 nCoV-19 vaccine, a viral vector vaccine. The patient also had an atypical presentation with a normal ESR and mildly raised CRP, which made the diagnosis difficult initially. He, unfortunately, developed permanent vision loss and became blind in the left eye. Considering there is a very short interval between vaccination and disease onset, it is possible vaccines may cause immune hyperreactivity leading to GCA. However, we can not confirm this with a case report, and chance association cannot be discarded. The lack of raised acute phase reaction can be misleading. GCA can be mistaken for vaccine-induced reactogenicity until an ischemic complication occurs. Further research is needed to confirm the relationship as causal or incidental to the possibility of the GCA post ChAdOx1 nCoV-19 vaccine and the likelihood of low-level inflammatory makers in such a situation.
